# Molecular basis for the plasticity of aromatic prenyltransferases in hapalindole biosynthesis

**DOI:** 10.3762/bjoc.15.157

**Published:** 2019-07-11

**Authors:** Takayoshi Awakawa, Ikuro Abe

**Affiliations:** 1 Graduate School of Pharmaceutical Sciences, The University of Tokyo, Bunkyo-ku, Tokyo 113-0033, Japan; 2 Collaborative Research Institute for Innovative Microbiology, The University of Tokyo, Yayoi 1-1-1, Bunkyo-ku, Tokyo 113-8657, Japan

**Keywords:** crystal structure, cyanobacteria, Friedel–Crafts reaction, hapalindole, prenyltransferase

## Abstract

Aromatic prenyltransferases (PTases) are enzymes that catalyze Friedel–Crafts reactions between aromatic compounds and isoprenoid diphosphates. In hapalindole biosynthesis, the aromatic PTases AmbP1 and AmbP3 exhibit surprisingly plastic selectivities. AmbP1 not only transfers the geranyl group on the C-3 of *cis*-indolylvinyl isonitrile, but also on the C-2, which is supressed in the presence of Mg^2+^ ions. AmbP3 transfers the dimethylallyl group on C-2 of hapalindole U in the reverse manner, but on C-2 of its C-10 stereoisomer in the normal manner. This review highlights the molecular bases of the AmbP1 and AmbP3 functions, elucidated through their X-ray crystal structures. The knowledge presented here will contribute to the understanding of aromatic PTase reactions and will enhance their uses as biocatalysts.

## Introduction

**Figure 1:**
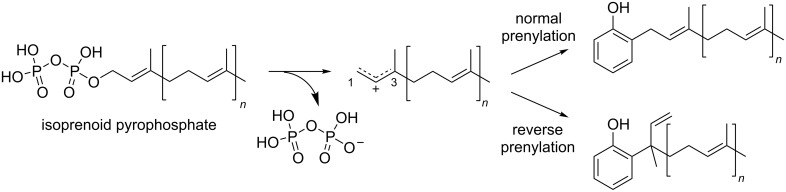
The reactions of aromatic PTases.

Aromatic prenyltransferases (PTases) catalyze Friedel–Crafts reactions between aromatic prenyl acceptors and isoprenoid diphosphate prenyl donors to construct C–C, C–O, or C–N bonds, which enrich the structural diversity of aromatic natural products [1–2]. Their reactions are divided into two types, depending on where the cation is generated in the isoprenoid diphosphate: the “normal” prenylation in which the C-1 is attacked and the “reverse” prenylation in which the C-3 is attacked (Figure 1). It is important to study prenylation types for the chemoenzymatic synthesis of bioactive compounds, because the prenylated compounds exhibit better bioactivities due to their improved interactions with biological membranes [3]. The aromatic PTase superfamily involved in the secondary metabolism consists of the ABBA (α-β-β-α barrel)-type [4–5], the dimethylallyltryptophan synthase (DMATS)-type [6–7], and the membrane-bound type PTases [8–9]. Some of them exhibit broad substrate specificities and accept various aromatic compounds as prenyl acceptors. For example, NphB (also called Orf-2), the first reported ABBA-type PTase in naphtherpin biosynthesis, accepts several aromatic compounds, including dihydroxynaphthalenes, flavonoids, and resorcinols as prenyl acceptors, and C_10_ geranyl diphosphate (GPP) as a prenyl donor, to generate a variety of O- or C-prenylated aromatic compounds [4,10]. Some PTases accept multiple lengths of isoprenoid diphosphates, as exemplified by the ABBA-type PTase TleC, which accepts C_5_ to C_20_ isoprenoid diphosphates in the biosynthesis of teleocidin B [11]. More interestingly, some PTases change their regiospecificity according to the chain length of the isoprenoid diphosphate, as exemplified by the DMATS-type PTase AtaPT [12]. To get knowledge about the molecular bases and their functions, a number of PTases have been subjected to X-ray crystallographical analyses. It is important to compare the multiple X-ray crystal structures with each substrate for the various reactions, to understand their plasticity. Here, we summarize the molecular basis of the two ABBA type PTases, AmbP1 and AmbP3, which catalyze multiple reactions with different sets of substrates [13–14]. Their plasticities in the reactions were revealed by the X-ray crystal structures of their complexes with different substrates.

## Review

### Hapalindole/ambiguine biosynthesis

**Figure 2:**
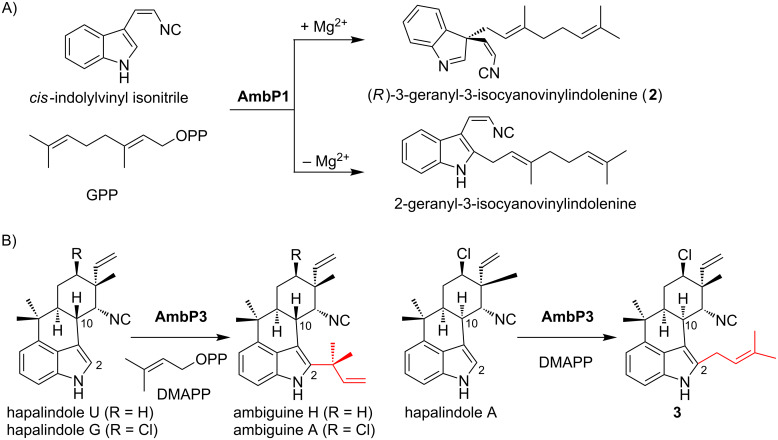
The reactions catalyzed by AmbP1 (A) and AmbP3 (B).

Hapalindole/ambiguine alkaloids, isolated from cyanobacteria, are composed of the total C_15_ prenyl moieties derived from dimethylallyl diphosphate (DMAPP) and geranyl diphosphate (GPP), and *cis*-indolylvinyl isonitrile **1** (Figure 2A) [15]. This natural product family includes structurally diverse compounds with beneficial bioactivities, exemplified by 12-*epi*-hapalindole E isonitrile, which exhibits antibacterial, antifungal, and antimycobacterial activities [16], and ambiguine I, which induces apoptosis and cell-cycle arrest through the inhibition of an NF-κB-related regulation pathway [17]. To investigate their biosyntheses, two research groups independently sequenced the genome of a cyanobacterium, *Fischerella ambigua* UTEX 1903, and performed a biosynthetic study [18–19]. Among the identified biosynthetic enzymes, the major contributors of the structural diversity are two prenyltransferases (AmbP1 and AmbP3) [18,20], isomerocyclases [19,21–24], and α-ketoglutarate-dependent oxygenases [25–26]. The prenyltransferase AmbP1 transfers a geranyl group onto C-3 of **1** to yield (*R*)-3-geranyl-3-isocyanovinylindolenine (**2**, Figure 2A) [20]. **2** is cyclized by isomerocyclases to give the hapalindole or fischerindole tetracyclic core structure [19,21–24]. The tetracyclic core is oxidatively halogenated by an α-ketoglutarate-dependent oxygenase [25–26]. Interestingly, AmbP1 also transfers the geranyl group onto the C-2 carbon of **1** to give *cis*-2-geranylindolylvinyl isonitrile, but this undesired side reaction is suppressed in the presence of Mg^2+^ (Figure 2A) [20]. AmbP3 exhibits tolerant substrate specificity with hapalindole substrates [18]. AmbP3 accepts (10*R*)-hapalindole U (HU) and G, and transfers the dimethylallyl group in the reverse prenylation mode to give ambiguine H and A, respectively (Figure 2B). Remarkably, AmbP3 also accepts (10*S*)-hapalindole A (HA), and transfers the dimethylallyl group onto the C-2 carbon of hapalindole A in normal prenylation mode to yield compound **3** (Figure 2B).

### X-ray crystal structure analysis of AmbP1

**Figure 3:**
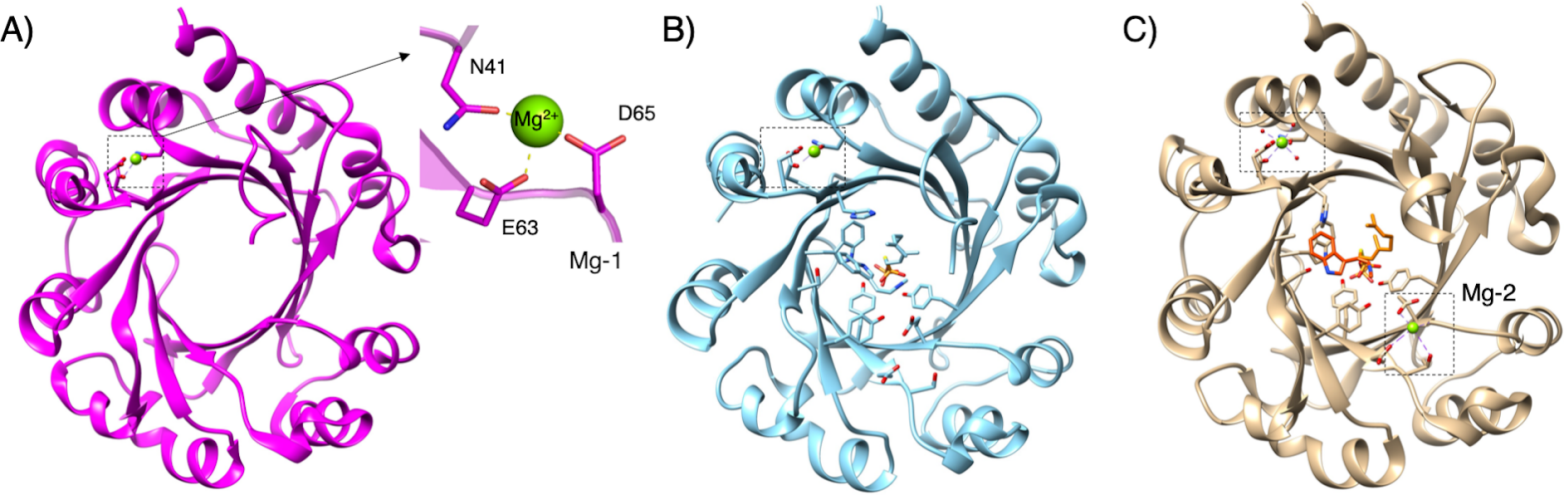
The overall structure of *apo*-AmbP1 (A), the Mg^2+^-free structure (B), and the Mg^2+^-bound structure (C).

**Figure 4:**
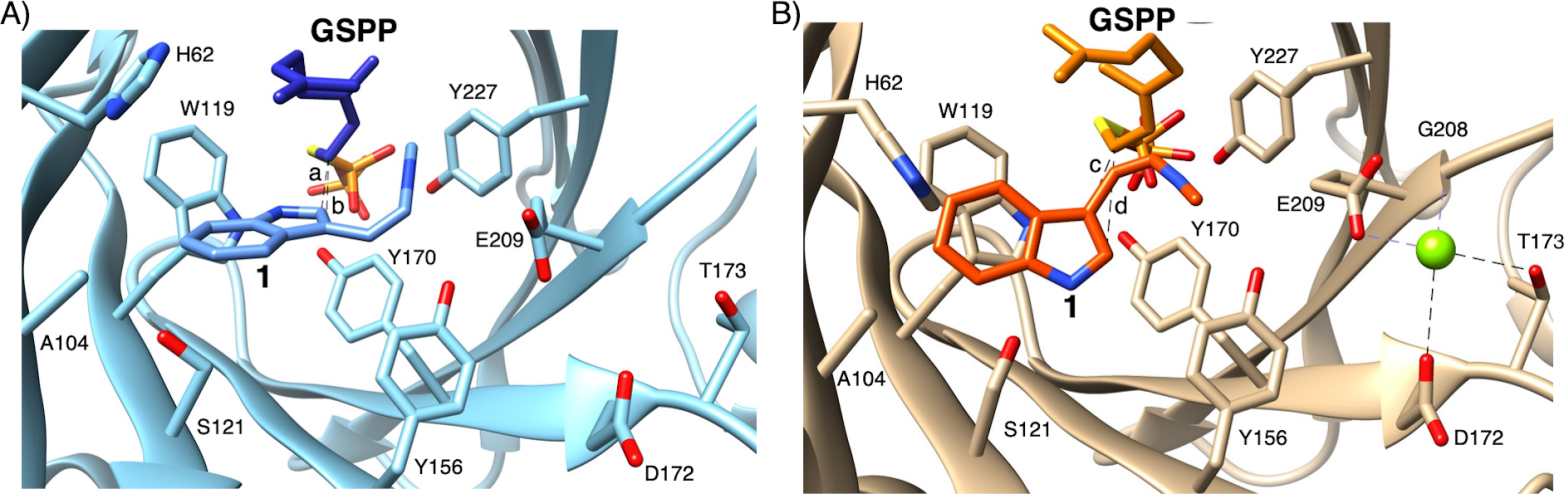
The active site structure of AmbP1. **1** and GSPP were bound in the active site without Mg^2+^ (A, Mg^2+^-free structure) and with Mg^2+^ (B, Mg^2+^-bound structure). The green sphere indicates the Mg^2+^ ion.

To understand the effect of Mg^2+^ ions on the AmbP1 reaction, an X-ray crystallization analysis was conducted. The *apo* structure of AmbP1 was solved at 2.35 Å, and it adopted an ABBA fold [4–5,13]. Interestingly, the *apo* structure unusually includes the Mg^2+^ ion in a position nearby the β-barrel, stabilized by hydrogen bonding with N41, E63, and D65 (Figure 3A, Mg-1). It might be required for structural integrity, although no mutational study has been performed to support this notion. In order to obtain a structure that is in complex with a substrate, **1** and geranyl *S*-thiodiphosphate (GSPP) were soaked into the crystal of AmbP1 at pH 6.5, which is the same pH as in the reservoir for crystallization. In this structure (Mg^2+^-free structure), the distance from C-1 of GSPP to C-2 of **1** (Figure 4A, **a**: 3.3 Å) is closer than that to C-3 of **1** (Figure 4A, **b**: 4.6 Å), unexpectedly indicating that this is a model for the C-2 prenylation. Next, the soaking experiment was tested at pH 8. Given that the suppression of C-2 prenylation in presence of Mg^2+^ ions is more obvious at pH 8–9 than pH 6 [20], it is expected that this soaking condition will provide the structural model of C-3 prenylation. As expected, the position of **1** dramatically changed, and the distance from C-1 of GSPP to C-3 of **1** (Figure 4B, **c**: 4.6 Å) became closer than that to C-2 of **1** (Figure 4B, **d**: 5.4 Å). Importantly, an additional Mg^2+^ ion (Figure 3C, Mg-2) appeared in the active site close to the isonitrile of **1**, stabilized by the hydrogen bonding with D172, T173, G208, and E209 (Figure 4B). The AmbP1 E209A and E209L mutants completely lost their activities, implying that E209 plays an important role in forming the catalytic cavity as well as binding the Mg^2+^ ion. The active site structure depicted by the surface mode indicated that E209 is important to form the wall of the cavity [13]. More interestingly, the D172A mutation altered the AmbP1 reaction, as it prefers C-3 prenylation even in the presence of Mg^2+^ ions. There are several X-ray crystal structural analyses of PTases that utilize Mg^2+^ as a Lewis acid, such as NphB [4], but this is the first structural analysis of the PTases that utilize a Mg^2+^ ion to reorganise the active site cavity to control the regiospecificity of the prenylation reaction.

### X-ray crystal structure analysis of AmbP3

**Figure 5:**
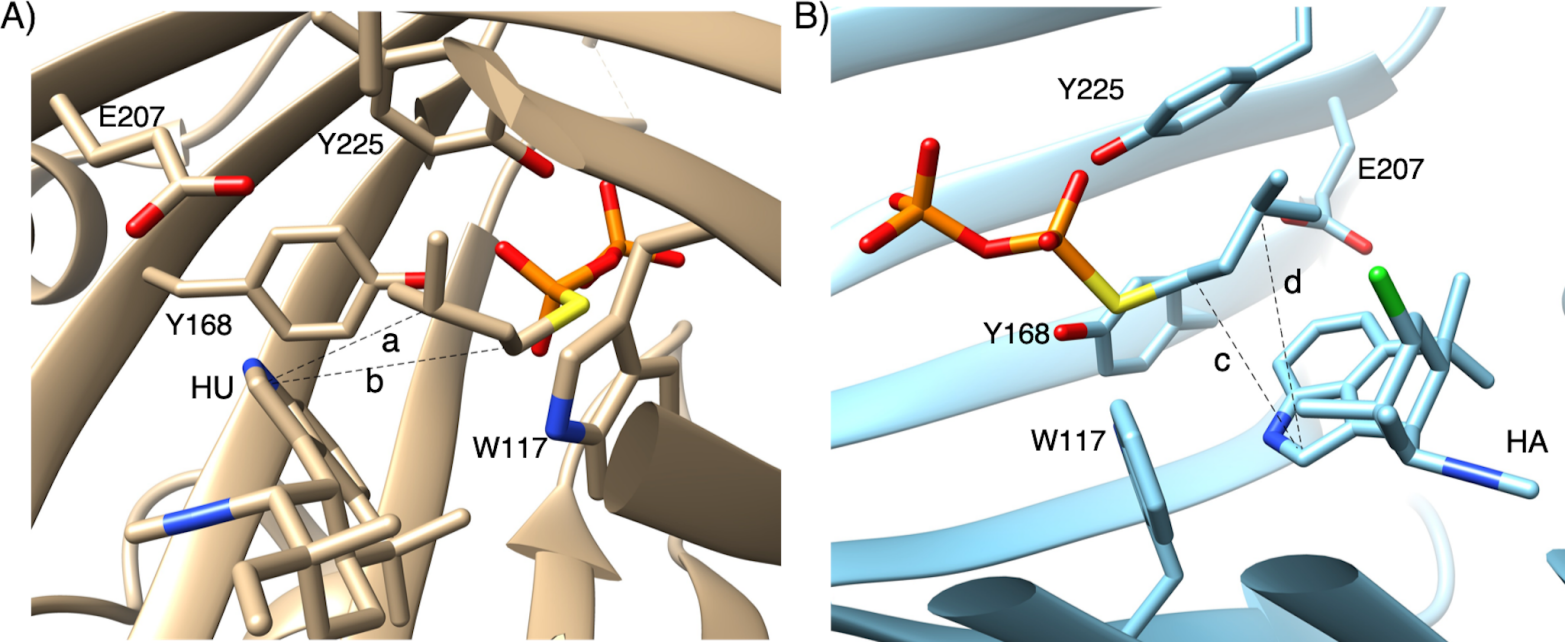
The active site structure of AmbP3 with substrates. The AmbP3 structure in complex with hapalindole U and DMSPP (A), and the AmbP3 structure bound in complex with hapalindole A and DMSPP (B).

The crystal structures of AmbP3 complexed with DMSPP/hapalindole U (HU structure) and A (HA structure) were each solved at 2.00 Å [14]. The prenyl acceptors, HU and HA, were both surrounded by hydrophobic amino acids, including A44, A102, W117, L119, L259, V284, F288, and M291 [14], and the position of HU was additionally stabilized by hydrogen bonding between the N-1 of HU and E207 (Figure 5A). Remarkably, the terpenoid moieties of HU and HA were located at the same position, but their orientations were completely different. These data indicated that the hydrophobic interaction between the enzyme and the terpenoid moiety is important to support the prenyl acceptor, and the orientation can be altered dependently on their steric structures. The indole of HU, W117, and Y168 formed a cation shield [27–28], which stabilizes the cation intermediate after the removal of the phosphate from DMAPP in the HU structure, and Y225 was substituted with Y168 in the HA structure (Figure 5). The orientation of W117 changed in accordance with the orientation of the indole in HU and HA. As expectedly, W117 was shown to be important for the reaction through a point mutation study, in which W117A and W117F completely lost the catalytic activity. In the HU structure, the distance between C-2 of HU and C-3 of DMSPP (Figure 5A, **a**: 3.6 Å) is shorter than that between C-2 of HU and C-1 of DMSPP (Figure 5A, **b**: 5.4 Å). On the other hand, in the HA structure, the distance between C-2 of HA and C-1 of DMSPP (Figure 5B, **c**: 4.6 Å) is shorter than that between C-2 of HA and C-3 of DMSPP (Figure 5B, **d**: 5.8 Å). These data are consistent with the preference of reverse prenylation on HU and normal prenylation on HA. This is the first X-ray structural model of a PTase that catalyzes both normal and reverse prenylations. The hydrophobic nature of the substrate-binding pocket and the flexibility of the amino acids shielding a cation lead to the plasticity to accept two stereoisomeric hapalindoles and DMAPP, in two different binding poses. This example also illustrates the plasticity of the PTase in the hapalindole biosynthesis. As described above, AtaPT accepts various aromatic compounds as prenyl acceptors, and changes the regiospecificity dependently on the prenyl donor [12]. This plasticity is due to the hydrophobic nature of the prenyl acceptor binding site and the fluctuations of the amino acids forming the cation shield, similarly to AmbP3.

### Comparison of the AmbP1 and AmbP3 amino acid sequences with other ABBA PTases

**Figure 6:**
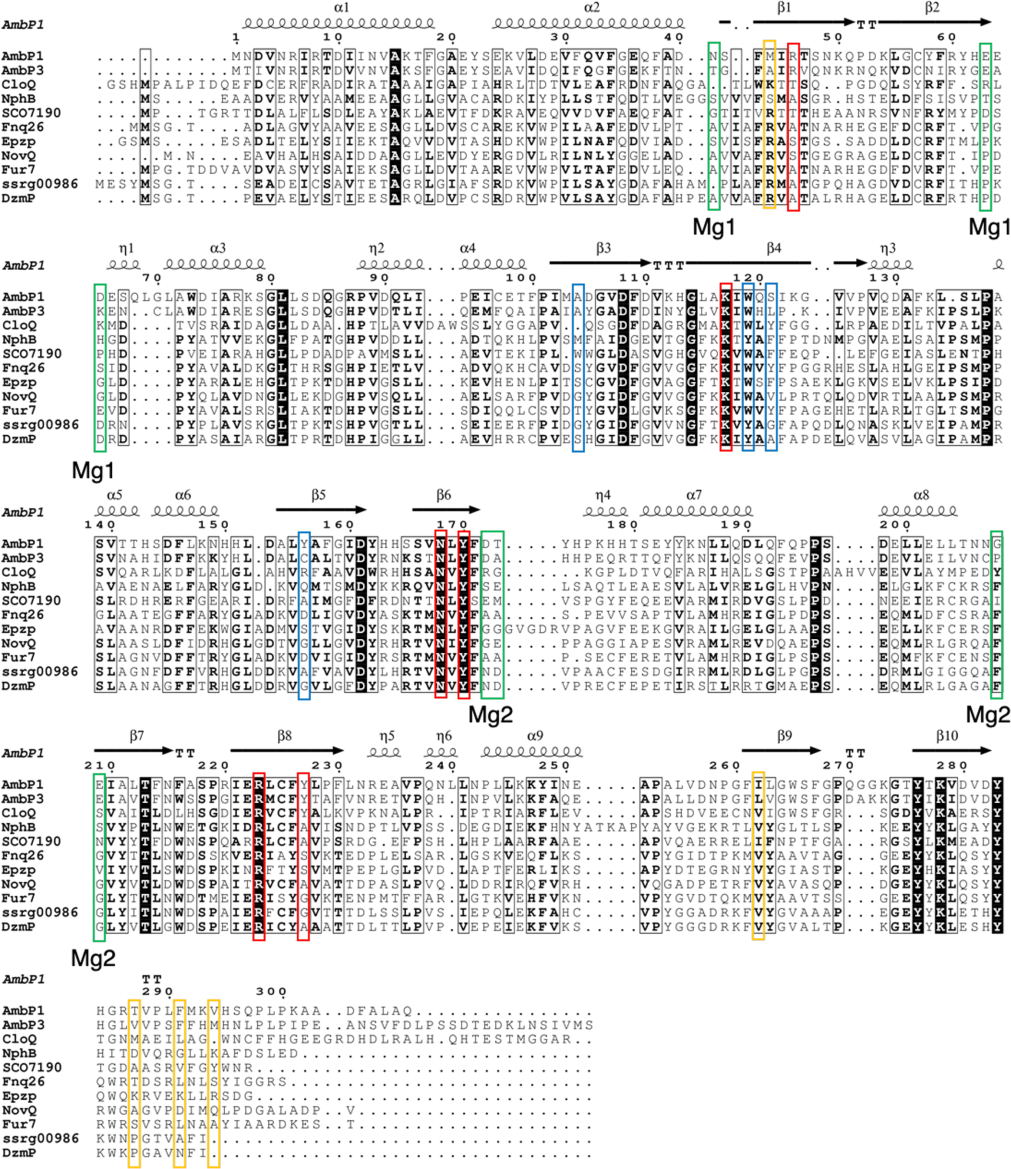
Multiple amino acid sequence alignment of AmbP1, AmbP3, and other ABBA PTases, visualized by ESPript3 [34]. Secondary structure elements: α, α-helices; β, β-strands; η, 3_10_-helices; TT, strict β-turns. The red frames indicate amino acids that anchor pyrophosphate, the blue frames indicate the residues that support the prenyl acceptor in AmbP1, and the yellow frames indicate the residues that support the prenyl acceptor for AmbP3 that are not conserved in AmbP1, and the green frames indicate the residues that anchor the Mg^2+^ ion.

The AmbP1 and AmbP3 amino acid sequences were aligned with the other ABBA PTases, including NphB [4], CloQ [28], SCO7190 [10], Fnq26 [29], EpzP [30], NovQ [31], Fur7 [32], SSRG00986 [33], and DzmP [33] (Figure 6). Most of the amino acids involved in pyrophosphate binding, including K117, N168, Y170, R223, Y227, Y276, and K278 in AmbP1, are well conserved among the ABBA family enzymes, but R46 is only conserved between AmbP1 and AmbP3, and Y227 is only conserved among AmbP1, AmbP3, and CloQ [28] (Figure 6), indicating that the structures for the pyrophosphate binding pockets in AmbP1 and AmbP3 are slightly different from those of the other enzymes. In fact, the position of the α-phosphate shifts between the Mg^2+^-free and -bound structures in AmbP1 and between the HU and HA structures in AmbP3, which alters the locations of the substrates in the enzyme. In the AmbP3 structures, Y225 plays an important role to form a cation shield in the HA structure, and the flexibility of Y225 is also an important factor to support both the prenyl acceptor and donor. These observations suggest that the binding mode of the pyrophosphate in AmbP1 and AmbP3 is unique in PTases, and it is likely to be the reason that allows the alternative binding modes of the substrates. The hydrophobic nature of the amino acids that support the prenyl acceptor should be a major factor to allow the alternative substrate binding modes for AmbP1 and AmbP3. Most of the hydrophobic amino acids that support the prenyl acceptor are not conserved among AmbP1, AmbP3, and the other PTases, but W119 is conserved or substituted with tyrosine among all of the aligned PTases (Figure 6). The aromatic amino acids around W119 are also important to support the aromatic substrates and cationic intermediates in the X-ray structural studies of CloQ and EpzP [28,30]. The orientation of W119 significantly changes in accordance with the substrate binding in the HU and HA structures of AmbP3, indicating that the flexible orientation of W119 is also an essential factor for the plasticity. Both two Mg^2+^ binding sites in AmbP1 are not conserved in the other PTases, indicating that they are a unique property of AmbP1. Remarkably, the two Mg^2+^- binding amino acids are located at the start or end of a β-sheet (Figure 6), which causes the corresponding β-sheet to move through the metal binding. Mg-1 is likely to maintain the overall structure of the enzyme, and Mg-2 defines the shape of the substrate binding site.

## Conclusion

The multiple structures of AmbP1 and AmbP3 with different substrate sets provide useful knowledge to understand the molecular basis of the promiscuous PTases. Their promiscuity is mainly caused by the hydrophobic binding pocket for the prenyl acceptor and the flexible positioning of the aromatic residues, which form a cation shield. Although recent progress in chemical synthetic research has established efficient ways to control normal and reverse prenylations with transition metal hydrides [35], it is still important to study the PTases, as they are useful catalysts that control the regiospecificity in an environmentally friendly manner. The information from their X-ray structures will contribute to future engineering of PTases. Furthermore, the structure of AmbP1 can serve as a model to alter the reaction through creating a metal binding site within the PTases, as the natural metalloprotein has been utilized as a model to create an artificial metalloprotein [36–37]. The increasing knowledge obtained from the X-ray structural studies of the PTases will contribute to the development of the enzymology and the chemoenzymatic syntheses of bioactive compounds.
